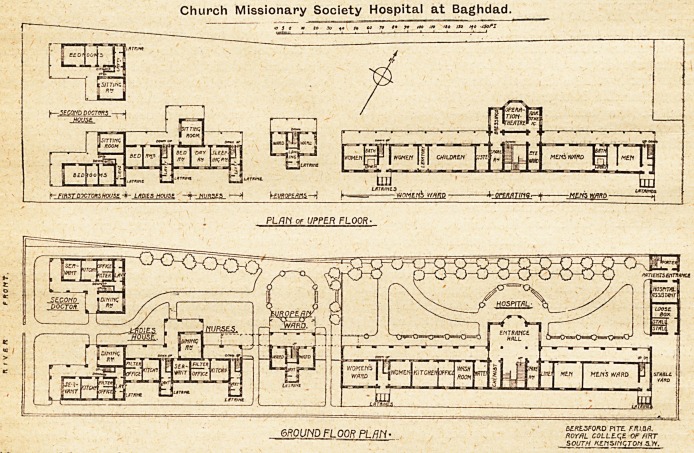# English Mission Hospital, Baghdad

**Published:** 1918-04-27

**Authors:** 


### ENGLISH MISSION HOSPITAL, BAGHDAD.

The new hospital was planned in 1912 for a long site bounded at the ends, to the S.W. by the River Tigris and to the N.E. by the road leading from the city. The requirements of the Mission included residences for two medical officers, a ladies' and nurses' house, a small block of private wards for European patients, besides the general hospital building for males and females, and the porter's house and stable.

The arrangement adopted places the hospital itself with convenient access from the road, the European ward beyond, and the residences at the private end of the site overlooking the river. The buildings are of two storeys with deep verandahs, excessive mid-day heat having to be reckoned with. The large wards are provided with wide verandahs to take couches on the front overlooking the gardens. The lavatories are disconnected from the main blocks on the back front. The construction is of stone, with girder and stone floors and roofs. The general scheme has been designed and settled in England, and in the entire absence of architectural detail is being carried out locally. The main block was erected as far as the roof at the outbreak of the war, and work will now be resumed. The main principle was the arrangement of the buildings on the site to secure the pleasantest use of the ground and adjustment to the prevailing wind so that the through current past the buildings was not obstructed. The scheme of the blocks and the connecting verandah will secure a dignified and intelligent arrangement without the requirement of costly or unsuitable architectural features.

The task of criticising plans of a hospital in a country where the conditions are so different from those which obtain in Europe is a difficult one; but, after all, a hospital must, in some particulars at all events, be arranged in much the same way whether it is at Baghdad or London. For example, for every large ward or group of small wards something in the nature of a ward kitchen must be required; a room where special diets can be prepared, where the ward crockery can be cleansed and stored and so forth. In the plan of the patients' building on the upper floor there is nothing of this kind; and on the ground floor there is only the kitchen to serve all purposes. The use of the room marked "office" next the kitchen is not clear, neither is that of the "wash-room."

Church Missionary Society Hospital at Baghdad.

In Baghdad there is no public water supply, and the inhabitants are dependent on the Tigris and what can be collected in rain-water tanks. Each household has therefore to filter its own water, hence the rooms marked "filter." In each of these there are four filters, and each block is supplied with its own filter-room; except apparently the European ward block. It may be, however, that water is carried from the filter-room in the adjoining block.

The European block seems to be planned on an irreducible minimum. There is no bathroom nor any nurses' room or ward kitchen—just two rooms on each floor with a latrine at the back.

## Figures and Tables

**Figure f1:**